# Feeling manipulated: cytomegalovirus immune manipulation

**DOI:** 10.1186/1743-422X-6-4

**Published:** 2009-01-09

**Authors:** Mindy Miller-Kittrell, Tim E Sparer

**Affiliations:** 1 Department of Microbiology, University of Tennessee, 1414 Cumberland Ave., Knoxville, TN, USA

## Abstract

No one likes to feel like they have been manipulated, but in the case of cytomegalovirus (CMV) immune manipulation, we do not really have much choice. Whether you call it CMV immune modulation, manipulation, or evasion, the bottom line is that CMV alters the immune response in such a way to allow the establishment of latency with lifelong shedding. With millions of years of coevolution within their hosts, CMVs, like other herpesviruses, encode numerous proteins that can broadly influence the magnitude and quality of both innate and adaptive immune responses. These viral proteins include both homologues of host proteins, such as MHC class I or chemokine homologues, and proteins with little similarity to any other known proteins, such as the chemokine binding protein. Although a strong immune response is launched against CMV, these virally encoded proteins can interfere with the host's ability to efficiently recognize and clear virus, while others induce or alter specific immune responses to benefit viral replication or spread within the host. Modulation of host immunity allows survival of both the virus and the host. One way of describing it would be a kind of "mutually assured survival" (as opposed to MAD, Mutually Assured Destruction). Evaluation of this relationship provides important insights into the life cycle of CMV as well as a greater understanding of the complexity of the immune response to pathogens in general.

## Introduction

After an initial primary infection herpesviruses establish latency for the life of the host with periodic and spontaneous reactivation. The coevolution of herpesviruses along with their host allowed these viruses to evolve mechanisms to modulate the host immune response. While some virally encoded proteins facilitate immune evasion, interfering with the host's ability to efficiently recognize and clear virus, others induce or alter specific immune responses to benefit the viral life cycle. Among the herpesviruses, cytomegaloviruses (CMVs) encode the greatest number of genes committed to altering both innate and adaptive immune responses (Additional files 1 and 2).

### Innate Immune Responses

#### Complement Cascade

The complement system is composed of a number of plasma proteins that induce inflammatory mediators, opsonization of pathogens, and direct lysis of pathogens and infected cells. The binding of complement proteins to antibody-antigen complexes activates the classical complement pathway, bridging innate and humoral immune responses. The binding of complement proteins directly to the surface of pathogens or infected cells initiates the alternative pathway. Both pathways lead to a common activation of the C3 convertase and subsequent elicitation of chemoattractants (e.g. C5a), opsonizing factors (e.g. C3b) and the membrane attack complex. Antibody-mediated complement lysis is an important mechanism for elimination of virus-infected cells. Therefore, a number of herpesviruses encode complement regulatory proteins. Herpes simplex virus 1 (HSV-1) and Epstein Barr virus (EBV) both have homologues of regulators of complement activation (RCA) proteins, such as CD46 and CD55 [1]. Complement control proteins including CD46, CD55, and CD59 regulate and also inhibit various stages within the complement cascade [2]. While no CMV encoded RCA homologues have been identified, CMVs have evolved mechanisms for limiting complement activity. HCMV infected fibroblasts are resistant to complement lysis when treated with CMV-specific antibodies, which would be expected to induce the classical complement pathway [3]. Human CMV (HCMV) incorporates cellular CD55 and CD59 into its virion. The importance of these complement inhibitory proteins was demonstrated when antibodies against CD55 decreased HCMV replication in fibroblasts following incubation with complement components [4]. The decrease in viral titers in the presence of these antibodies showed that inhibition of complement activation (i.e. interfering with inhibitory CD55) is important for HCMV replication. HCMV also upregulates the expression of CD46 and CD55 on the surface of infected cells, which decreases the accumulation of C3 convertases. This in turn, protects the cells from complement mediated lysis [5]. Although the mechanisms differ from other herpesviruses, HCMV is able to inactivate the complement cascade, increasing virus replication/survival.

Mouse CMV (MCMV) also induces CD46 expression on infected fibroblasts. This upregulation was mapped to a CMV responsive element within the CD46 promoter [6]. Mice typically express the complement regulator CRRY instead of CD46 [7]. However, MCMV infected cells resist complement lysis and CD46 expression is associated with this decrease [6]. The viral proteins that interfere with this specific, antibody-mediated complement activity during MCMV infection have not been identified.

#### Fc Receptor Homologues

Clinical evidence supports a role for antibodies in limiting cytomegalovirus infection [8] and modulation of antibody-mediated immunity would be beneficial for CMV survival. CMV infected cells can bind immunoglobulins in serum of seronegative individuals [9-12]. This phenomenon was linked to the expression of Fc binding proteins (FcBPs) on the surface of infected cells that are specific for IgG but not other isotypes [13-15]. Both MCMV and HCMV encode FcBPs. MCMV encodes one FcBP, fcr-1 or m138. Although, m138 has no homology to the HCMV Fcγ BP it has conserved structural features with cellular Fcγ receptors (FcγRs) and the viral FcγR of HSV (gE-gI) [16]. Normally the FcRs from the host interact with the Fc region of Igs and mediate a number of effector functions including phagocytosis, release of inflammatory cytokines, and natural killer (NK) cell-mediated antibody dependent cellular cytotoxicity (ADCC)[17]. It has been speculated that the Fc region of anti-CMV antibodies bind the viral FcγR instead of host FcγRs and block antiviral activities. m138 is important for MCMV replication *in vivo*. However, both wild-type and B cell deficient mice infected with a Δm138 MCMV mutant had lower viral titers in several organs compared to mice infected with wild-type MCMV. Thus impaired growth of MCMV is not due to an enhanced humoral immune response in the absence of m138 (i.e. similar titers were found in the presence or absence of humoral responses) [18,19]. Interestingly, m138 downregulates expression of H60 and MULT-1, two ligands of the activating NK receptor, NKG2D. The importance of this downregulation *in vivo *was demonstrated when the Δm138 MCMV was found at two logs reduced titer compared to wt MCMV. Both replicated equivalently in NK cell depleted mice. The downregulation of MULT-1, but not H60, is due to defective cellular trafficking that results in degradation of the protein [20]. m138 also decreases cell surface trafficking of the costimulatory molecule B7-1 on dendritic cells (DCs), interfering with the ability of these cells to activate antigen specific T cells [21]. Thus the MCMV FcγR should broadly affect both NK cell recognition and perhaps presentation to the adaptive immune response, although in a manner different from HSV-1 gE-gI.

Two HCMV FcγRs have been identified, gp34 and gp68, which are encoded by the genes *TRL11/IRL11 *and *UL119-UL118*, respectively [22,23]. Although the function of the HCMV-encoded FcγRs has not been determined, initially it was assumed they would function like the viral FcγR from HSV-1 (gE-gI), which protects infected cells from ADCC and complement activation [24,25]. Recently it was shown that gp68 binds the same IgG domain and with the same stoichiometry as HSV-1 gE-gI. However, the two proteins have different pH requirements for binding which may relate to different mechanisms following IgG engagement, although this possibility requires additional study [26].

#### Interferon-Mediated Immunity

While interferons (IFNs) are induced early during CMV infection [27-32], CMVs encode a number of proteins that mitigate the effects of IFN activation [33-35]. The type I interferons IFNα and IFNβ are part of the innate immune response to viral infections [36]. The binding of IFNα and IFNβ to the IFN receptor induces Jak/Stat signalling leading to the rapid upregulation of interferon-stimulated genes (ISGs), such as MHC class I molecules and various cytokines [37]. Two HCMV immediate early gene products, 72 kDa immediate early 1 (IE1) protein (IE1-p72) and IE2 protein (IE86), interfere with IFN signalling. IE1-p72 binds STAT2 inhibiting the ISG transcriptional activator, ISGF3, from binding to the ISG promoter [38,39]. IE86 blocks binding of the transcription factor NFκB to the IFNβ promoter and leads to the downregulation of IFNβ expression in HCMV infected fibroblasts [40,41].

The HCMV tegument protein pp65 (*UL83*) has been implicated in blocking interferon activity early during infection prior to transcription of the immediate early gene products [30]. Microarray analysis using wt HCMV and a *UL83 *deletion mutant revealed pp65 downregulates numerous ISGs although the mechanism for this is unclear [42]. Conflicting reports have attributed this decrease to interference with either NFκB activity or another transcriptional regulator, IRF-3, although both authors suggest a pp65-mediated defect in nuclear localization of the transcription factors likely contributes to the decreased activity [42,43]. However, a *UL83 *mutant virus like that used in these previous studies was also shown to have decreased IE86 expression. In contrast, cells infected with an HCMV mutant in which stop codons were used to disrupt pp65 expression, preserving IE86 expression, showed no difference in IFN expression compared to wt HCMV [44]. This suggests IE86 activity is crucial in mitigating IFN-mediated events following infection. Nevertheless, the conservation of *UL83 *within CMV genomes and the attenuation of an MCMV lacking expression of the murine pp65 homolog [45] points to the importance of pp65 for CMV survival *in vivo*.

The MCMV encoded protein, M27, interferes with both type I and type II (IFNγ) interferon activity by downregulating the signalling molecule, STAT2 [46,47]. M27 decreases transcription of constituents of the immunoproteasome [48]. This IFNγ inducible proteasome enhances MHC class I processing of peptides for presentation. Therefore, M27 may not only protect MCMV from the direct antiviral effects of interferon induction but also interfere with the presentation of viral peptides to CD8^+ ^T cells, a common theme to be discussed below.

MCMV and HCMV also encode proteins that interact with interferon induced gene products. The MCMV gene products m142 and m143 decrease activation of protein kinase R (PKR) [49], an enzyme that blocks protein synthesis in infected cells leading to impaired viral replication. PKR is activated via dsRNA, which is produced during transcription of complementary strands of the CMV genome. Both m142 and m143 have dsRNA binding domains and may interfere with the binding of PKR with dsRNA, preventing its activation [50]. The HCMV encoded proteins TRS1 and IRS1 are capable of binding dsRNA and can substitute for the vaccinia virus (VV) RNA binding protein [51,52]. However, the role of these proteins in the context of HMCV infection has not been evaluated [53].

#### Natural Killer Cells

Natural killer (NK) cells are cytotoxic cells of the innate immune response that play an important role in eliminating virus-infected cells early during infection. Signalling induced via activating receptors in absence of inhibitory receptor signalling regulates the cytotoxic activity of NK cells. Inhibitory receptors recognize specific MHC class I alleles whereas a number of ligands can bind activating receptors to trigger NK cell mediated killing. NK cells are important for controlling CMV infections both in mice and humans [54]. Therefore, it is not surprising the CMVs encode numerous proteins that interfere with NK cell activity [55].

Some HCMV encoded proteins alter expression of NK cell receptor ligands. The *UL16 *encoded glycoprotein, gpUL16, binds a number of ligands of the NK cell activating receptor, NKG2D. gpUL16 binds members of the UL16-binding protein (ULBP) and MIC family of proteins, which are stress induced NK ligands [56]. In particular, gpUL16 binds to ULBPs 1 and 2 [57,58] and MICB [59]. gpUL16 downregulates these proteins, retaining them intracellularly in the ER or Golgi network [60-62]. The interaction of gpUL16 with these ligands blocks NK cell activation *in vitro *[56,57,63,64] and may represent a way for HCMV to inhibit NK cell activation due to replication-induced cell stress.

Like gpUL16, the recently identified HCMV microRNA (miRNA), miR-UL112, also decreases expression of the NK ligand, MICB. miRNAs bind to 3' UTRs and prevent their translation. The miR-UL112 mediated decrease in MICB helps protect HCMV-infected cells from NK cell recognition and killing [65] and suggests that control of MICB expression and avoidance of NK recognition is important to the HCMV life cycle. As the field of CMV miRNA's begins to expand, an understanding of the role that miRNAs play in immune manipulation will also expand.

MCMV also encodes three proteins that downregulate ligands of the NK cell activating receptor, NKG2D. The *m145 *encoded protein decreases expression of MULT-1 [66], the *m155 *encoded glycoprotein (m155) downregulates H60 [67], and the *m152 *encoded glycoprotein (gp40) downregulates proteins of the RAE-1 family [68-70]. MCMV mutants lacking any one of these genes are attenuated *in vivo *and NK cell depletion restores MCMV growth. These results support a role for these proteins in inhibiting NK cell mediated clearance [66,69,71,72]. Although the exact mechanism of action is not known for these proteins, both m155 and gp40 act at a post-transcriptional step and likely interfere with trafficking of NK ligands to the cell membrane [67,69,72].

MCMV encodes two homologues of MHC class I molecules, although neither has similarity to the HCMV homolog, *UL18 *[73,74]. The MCMV gene *m157 *has low sequence homology to MHC class I molecules but structural analysis shows it has MHC-like folds [75]. m157 is expressed on infected cells and is tethered to the membrane with a glycosylphosphatidylinositol (GPI) anchor [76,77]. m157 binds to Ly49H, an activating receptor of the Ly49 family of NK receptors [75,78]. Interestingly, the m157-Ly49H interaction activates NK cells *in vitro *inducing NK cell cytotoxicity and cytokine and chemokine expression [79,80]. In the structural paper exploring m157/Ly49H interactions, Adams et al showed that m157 activation of Ly49H is sufficient to override MHC I/NK cell inhibition [81]. *In vivo*, MCMV mutants lacking m157 are more virulent in mice due to decreased NK cell activity [82]. Furthermore, MCMV replicates to higher titers in mouse strains that lack expression of Ly49H receptors [83,84], demonstrating that Ly49H activation is important for immune protection against MCMV. Although Ly49H protects against MCMV in laboratory mice, MCMV isolates from wild mice have m157 variants that fail to activate NK cells, thus providing evidence of evasion of NK recognition [85]. Mutations in m157 were also shown to accumulate following multiple passages of MCMV in mice indicating selection pressure for m157 variants that fail to activate NK cells. Furthermore, m157 also binds the inhibitory NK receptor, Ly49I, of some susceptible mice strains such as 129/J [75]. This has led to the speculation that CMVs evolved m157 to interact with NK inhibitory receptors but in some strains of mice this immune manipulation "backfires" and causes NK cell activation. These data suggest that m157 activation of NK may occur less frequently in natural infection of mice in the wild.

*m144 *is another MCMV encoded MHC class I homolog [74,86]. m144 is expressed on the cell surface despite its inability to bind endogenous peptides like other MHC homologues [87]. m144 can inhibit NK cell cytolysis *in vitro *[88], suggesting it serves as a decoy receptor to inhibit NK cell activation. Consistent with this idea, MCMV mutants without m144 expression grow poorly in mice due to enhanced NK cell activation [89]. While a flexible region within m144 that could potentially interact with host receptors has been identified [90], m144 has not been shown to bind any cellular receptors and its mechanism of function remains unknown [88]. A rat CMV (RCMV) homolog of m144, r144, has also been identified [91]. Wild type RCMV shows enhanced replication in the salivary gland and spleen of neonatal rats when compared to an r144 deletion mutant [92]. However, what affect this protein has on NK cell activity is not currently known.

HCMV encodes several proteins that interact with and alter NK cell responses. One of the earliest identified proteins, gpUL18, is encoded by the gene *UL18 *and is a MHC class I homologue [93,94]. gpUL18 binds the β_2_-microglobulin (β_2_m) and unlike the MHC homologue of MCMV, m144, gpUL18 also binds endogenous peptide [95,96]. gpUL18 binds the NK cell inhibitory receptor LIR-1 with higher affinity than host MHC class I molecules [97-100]. However, the function of gpUL18 has not been clearly defined. gpUL18 expression both inhibits and activates NK cells *in vitro *[101-106]. For instance, gpUL18 activates NK cells from LIR-1^- ^mice implicating a complex mechanism for NK cell modulation. Furthermore, clinical isolates of HCMV express gpUL18 variants with different LIR-1 binding affinities [107,108] that may have differential functions. HCMV gpUL18-deletion mutants have shown both gpUL18 dependent and independent effects on NK cell activity in different cellular systems [101,109]. In addition to its effect on NK cells, gpUL18 was recently shown to inhibit dendritic cell maturation and migration. While dendritic cells express LIR-1, whether this receptor mediates the effect of gpUL18 on dendritic cell activity was not determined [110]. Importantly, the HCMV encoded proteins that downregulate host MHC class I molecules do not interfere with the expression of gpUL18, which provides "protection" from NK cell lysis that occurs when MHC class I is downregulated [111,112]. Collectively, current data suggests gpUL18 may impact the function of several cell types *in vivo *that may partially relate to its ability to bind and activate LIR-1 [113].

Recently, it was found that the *UL142 *gene encodes a second HCMV MHC class I homologue [114]. Cells expressing gpUL142 are protected from NK cell lysis. However, this effect was not evaluated in the context of HCMV infection [115]. While the mechanism of this effect has yet to be determined, gpUL142 was shown to downregulate MICA, a ligand for the activating NK cell receptor, NKG2D [116].

Interestingly, HCMV increases expression of the non-classical MHC class I molecule, HLA-E, while downregulating expression of many other MHC class I alleles [117]. This upregulation is due to the expression of UL40, a HCMV encoded protein that contains a peptide sequence identical to the HLA-E leader peptide [118-120]. Therefore, UL40 increases expression of HLA-E independent of TAP mediated peptide processing, which the HCMV encoded protein, gpUS6, inhibits during infection [121]. HLA-E is a ligand for the inhibitory receptor CD94/NKG2A and the UL40 induced upregulation of HLA-E would be expected to protect HCMV infected cells from NK cell lysis. However, conflicting reports have found UL40 inhibited or had no effect on NK cell lysis of HCMV infected cells [117,122]. Additionally, the HLA-E restricted NK-CTLs recognize the UL40 peptide and kill virally infected cells [123,124]. Thus, whether UL40 protects HCMV infected cells or targets them for destruction requires further evaluation.

The tegument protein, pp65, also interferes with NK cell function by binding the activating receptor NKp30. pp65 mediates the dissociation of CD3ξ, a signal transducing polypeptide, from NK cells impairing their activation [125]. Since pp65 is not secreted or expressed on infected cells, it is currently unclear how this protein mediates its effects.

Finally, the protein product of *UL141*, gp141, decreases the expression of CD155, a ligand for NK cell activating receptors by inhibiting CD155 transport to the cell surface [126]. This downregulation inhibits NK cell activity *in vitro *but further information regarding its function and impact *in vivo *remain to be answered.

#### Cytokine Homologues

Interleukin-10 (IL-10) is an immunosuppressive cytokine that downregulates inflammatory cytokine synthesis and interferes with antigen presentation by decreasing expression of MHC class II on antigen presenting cells [127]. Members of the poxvirus and herpesvirus families, including HCMV, encode IL-10 homologues [128]. In contrast to other viral IL-10 homologues, such as those encoded by EBV and orf virus, a poxvirus, both of which have high amino acid similarity (80%) to their host IL-10. The HCMV IL-10 protein, cmvIL-10, has only limited homology (27%) to human IL-10 (hIL-10) [129]. cmvIL-10 binds the IL-10 receptor, hIL-10R, albeit with lower affinity than hIL-10 [129,130]. Nevertheless, it retains the capacity to induce a strong anti-inflammatory response. cmvIL-10 downregulates expression of IFNγ and TNFα in peripheral blood mononuclear cells (PBMCs) and decreases expression of both MHC class I and II on PBMCs and DCs [130-133]. DCs exposed to cmvIL-10 inefficiently stimulated T cell proliferation [132] supporting its role in immune suppression. Furthermore, cmvIL-10 inhibits cytokine production due to the activation of the phosphotidylinositol 3-kinase pathway [134]. Interestingly, cmvIL-10 was recently shown to stimulate B cell proliferation but the impact of this on the immune response to CMV is unclear [135]. cmvIL-10 alters the function of non-leukocyte populations as well. By interfering with placental cytotrophoblast invasion, cmvIL-10 could affect placental development, which contributes to the sequelae observed following congenital CMV infections [136].

An alternatively spliced version of cmvIL-10 was originally identified during latent HCMV infection and termed latency associated cmvIL-10 (LacmvIL-10) [137]. However, this transcript is also expressed during productive infection [138]. LacmvIL-10 fails to induce signalling pathways associated with IL-10R activation, which implies LacmvIL-10 may not bind to IL-10R or, at least, differentially activates this receptor. LacmvIL-10 also decreases MHC class II expression on granulocyte-macrophage progenitors (GM-Ps) and monocytes, both sites of CMV latency, possibly limiting clearance of latently infected cells [139].

A number of primate CMVs encode proteins similar to IL-10 [140] but only the function of the rhesus CMV (RhCMV) homologue has been evaluated to date. RhcmvIL-10 downregulates cytokine expression in PBMCs and MHC class II molecule expression in monocytes [130]. Therefore, the function of human cmvIL-10 is conserved in RhCMV. Interestingly, chimpanzee cytomegalovirus (CCMV) and MCMV do not encode IL-10 homologues [140]. MCMV infection induces cellular IL-10 expression providing a mechanism for immune interference in the absence of a virally encoded IL-10 protein [141,142]. Therefore, IL-10 immune suppression, whether cellular or viral in origin, likely creates an environment that supports efficient viral replication in the host.

#### Cytokine Receptor Homologues

The HCMV gene, *UL144*, encodes a protein with limited homology to the herpes simplex virus entry mediator (HVEM), a member of the tumour necrosis factor receptor (TNFR) superfamily [143]. UL144 is the only TNFR homolog identified in herpesviruses. The UL144 protein does not bind any known TNF ligands [143-145]. UL144 binds to a member of the Ig superfamily, B and T lymphocyte attenuator (BTLA) [146], also a ligand of HVEM [147]. Binding of UL144 to BTLA blocks T cell proliferation and could impair lymphocyte responses to HCMV [146]. UL144 activates TNFR-activated factor (TRAF6) leading to NFκB activation and upregulation of the chemokine CCL22 [145,148]. CCL22 is a chemoattractant of Th2 and regulatory T cells. Activation and attraction of these cells may help HMCV evade T cell-mediated antiviral activity. UL144 is located in the UL/b' region of HCMV, a portion of the genome thought to encode potential virulence factors. Different UL144 genotypes have been identified in clinical isolates. However, all but one report has found no association between the different genotypes and CMV disease [144,149-155]. Although there is much speculation on the role that UL144 plays in immune modulation, its role in CMV pathogenesis and survival is still unclear.

#### Viral Chemokine Homologues

Many CMVs also encode chemokine homologues. Chemokines are small, chemotactic cytokines that are important for leukocyte trafficking and activation. The best characterized of the CMV chemokines is the CC chemokine homolog of MCMV. Originally identified as the *m131 *gene product, MCK1, transcriptional analysis during MCMV infection determined that a spliced product of *m131 *and *m129*, referred to as MCK2 was the only transcript encoded from this locus. The CC chemokine domain is confined to the MCK1 coding region and is connected to a long carboxyl-terminal domain (199 amino acids) with no known homology to other proteins. This makes MCK2 considerably longer than other chemokines [156,157]. In contrast to *UL146 *(see below), the DNA sequence of MCK2 is highly conserved in isolates from wild mice [158].

Initial studies *in vitro *showed MCK1 induced higher levels of Ca^2+ ^mobilization in peritoneal exudates cells from MCMV infected mice than cells from uninfected mice. This demonstrated that MCK1 could activate cells recruited to the site of MCMV infection [159]. The spliced product, MCK2, is important for viral spread within the host. When a recombinant MCMV with mutations in the *m131 *gene (generated either via point mutations or insertions) was inoculated into mice, these recombinants showed a defect in dissemination to the salivary gland. Mice infected with these mutants developed less inflammation at the site of inoculation, reduced secondary viremia, as well as lower viral titers in the salivary glands, a site of dissemination following secondary viremia [160,161]. This was the first direct evidence that a CMV encoded chemokine is important for the dissemination of the virus in its host. Additional work identified the MCK2-recruited cell type as a late myeloid progenitor, consistent with reports that CMV infects and can remain latent in cells of the myelomonocytic lineage [162].

Rat CMV (RCMV) encodes a CC chemokine with similarity to both *m131 *and *m129*, having N-terminal chemokine homology and a long carboxyl-terminus like MCK2 [163]. Rats infected with RCMV deletion mutants lacking pr131 expression, the product of the r131 locus, had reduced viral loads in the spleen and salivary glands. Additionally these mice had reduced swelling and macrophage infiltration at the site of virus inoculation [164]. Therefore, pr131 appears to be a functional homolog of MCK2.

Guinea pig CMV (GPCMV) contains a gene with homology to CC chemokines, though it lacks positional or sequence similarity to the *m131*/*m129 *genes of MCMV. GPCMV-MIP is most similar to the cellular chemokines, CCL3/CCL4 (MIP-1α/β) and CCL14 (HCC-1). GPCMV-MIP induced Ca^2+ ^flux and migration of cells expressing hCCR-1 but did not elicit a response from cells expressing other CC receptors [165]. CCL14 is known to increase the proliferation of monocyte progenitors, therefore GPCMV-MIP may enhance the proliferation and/or recruitment of permissive cells consistent with the role of MCK2 [166].

HCMV encodes two ORFs with homology to CXC chemokines, *UL146 *and *UL147 *[167]. While no functional data is available for the protein product of UL147, vCXCL-2 (also called pUL147 and vCXC-2), analysis of the protein product of *UL146*, vCXCL-1 (also denoted pUL146 and vCXC-1), demonstrated its capacity as a functional chemokine. vCXCL-1 was shown to bind exclusively to hCXCR2 with an affinity similar to that of the host chemokine, CXCL8. Additionally, vCXCL-1 was able to induce chemotaxis and intracellular calcium release in human neutrophils, again to levels comparable to host chemokines [168]. A mutant HCMV virus with a deletion of *UL146-UL147 *was unable to infect neutrophils but retained its ability to infect other cells providing additional evidence these proteins facilitate an interaction of HCMV with neutrophils [169].

Chimpanzee CMV (CCMV) encodes homologues of *UL146 *and *UL147 *as well as the related gene *UL146a*. However, only the product of *UL146 *(vCXCL-1_CCMV_) has been evaluated thus far. vCXCL-1_CCMV _induces chemotaxis and calcium release in human neutrophils. Additionally, it was shown to increase expression of adhesion molecules on the surface of neutrophils and reduce apoptosis in these cells [170]. Taken together, the function of the HCMV and CCMV vCXCL-1s *in vitro *suggest the potential for these viral chemokines to alter the response of neutrophils in the course of CMV infection.

Due to the strict species specificity of CMVs, *in vivo *characterization of *UL146 *and *UL147 *has been limited to sequence analysis of clinical HCMV isolates. *UL146 *is highly variable, differing as much as 60% among isolates at the amino acid level [152]. It was postulated that this variability might correlate with the severity of CMV disease however no clear relationship has been identified [151,171-173]. It was recently suggested the variability of *UL146 *may have arisen in early human populations and likely does not contribute to the pathogenesis of congenital infection [173]. Our lab is currently working to identify whether the vCXCL-1 isolates from different clinical strains induce functional differences in neutrophils.

HCMV also encodes genes with limited homology to CC chemokines. *UL128 *and *UL130 *both have signal sequences and conserved cysteine motifs similar to CC chemokines [174], but it is yet to be determined whether these proteins actually function as chemokines. Interestingly, the *UL128-131 *locus was found to be necessary for endothelial cell and leukocyte tropism based on the inability of HCMV *UL128-131 *deletion mutants to infect these cell types [169]. This likely explains the loss of endothelial cell tropism in lab-adapted strains of HCMV, a number of which were shown to have deletions or mutations in at least one gene from this region [174]. The UL128-131 proteins have recently been shown to interact with gH/gL complexes that mediate entry of CMV into endothelial and epithelial cells, in contrast to the gH/gL complexes that mediate virus entry into fibroblasts [175,176].

#### Chemokine Receptor Homologues

In addition to viral chemokine homologues, CMVs encode proteins with homology to chemokine receptors [177,178]. Chemokine receptors are members of the G-protein coupled receptor (GPCR) family [179]. The four chemokine receptor homologues encoded by the cytomegaloviruses have homology to the CC chemokine receptor family. Two genes, *UL33 *and *UL78*, are conserved among all sequenced CMVs, while primate CMVs encode two additional GPCRs, *US27 *and *US28 *[180].

The HCMV *US28 *encoded protein (pUS28) is the best characterized of the GPCR homologues. *US28 *has the highest sequence homology to the cellular receptor, CCR1, and binds a number of CC chemokines including CCL5 (RANTES), CCL2 (MCP-1), CCL3 (MIP-1α), and CCL4 (MIP-1β) [181,182]. Interestingly, pUS28 binds with highest affinity to the CX_3_C chemokine, CX_3_CL1 (fractalkine) [183]. Upon binding, pUS28 induces chemokine internalization, removing chemokines from the extracellular environment [184-186]. Consistent with this role, pUS28 inhibits monocyte migration *in vitro *and media from US28 expressing, CMV-infected monolayers deplete CCL5 and CCL2 from the media [187]. Constitutive endocytosis and recycling of pUS28 contributes to chemokine internalization however chemokine binding is not sufficient to induce constitutive endocytosis of pUS28 [188]. By acting as a "chemokine sink", pUS28 could alter or inhibit chemokine-dependent immune responses. The related chemokine homolog, pUS27, also shows some ability to internalize chemokines [185,189], perhaps illustrating a conservation of function between the viral GPCRs.

pUS28 elicits ligand-dependent signalling, triggering calcium mobilization upon CCL5, CCL2, CCL3 and CCL7 binding. pUS28 utilizes the G proteins Gα_i _and Gα_16 _to mediate calcium mobilization upon binding of CCL5 or CCL7 [181,190,191]. Interestingly, Gα_16 _expression is restricted to hematopoietic cells [192]. HCMV has a tropism for this cell lineage where pUS28 may alter Gα_16 _signalling to benefit CMV's life cycle. pUS28 also induces agonist-dependent migration of smooth muscle cells (SMCs). Migration of SMCs in response to pUS28 requires the protein tyrosine kinase (PTK) pathway, indicating pUS28 activates a number of cellular pathways *in vitro *[193,194]. As with many CMV proteins, the effects of pUS28 vary depending on the system and cell types analyzed.

Like the Kaposi's sarcoma-associated herpesvirus (KSHV) GPCR homolog, ORF74 [195], pUS28 can constitutively activate several signalling pathways, including phospholipase C (PLC), NFκB, NFAT, and cAMP-responsive element (CRE)-dependent pathways [188,191,195-198]. CC chemokines do not enhance signalling, although CX_3_CL1 acts as a partial inverse agonist, decreasing the levels of pUS28 constitutive activity [191,196]. The constitutive endocytosis of pUS28 occurs independently of its constitutive signalling and different domains of pUS28 control these two phenomena [188]. pUS28, like KSHV ORF74, can induce transformation of NIH3T3 cells and promote tumour formation in nude mice. Mice inoculated with cells expressing a mutated pUS28 that lacks constitutive activity show attenuated tumour formation demonstrating the importance of constitutive signalling in pUS28-mediated tumour formation [199].

Despite extensive research on the function of pUS28, its role in CMV pathogenesis *in vivo *is still unknown. pUS28 sequesters host chemokines in order to alter the immune response or activates signalling pathways that contribute to viral replication or spread. pUS28-induced SMC migration may play a role in the accelerated vascular disease associated with HCMV [200,201]. Although HCMV nucleic acids have been isolated from certain cancers [202,203], whether HCMV is causal in these cancers remains to be established. Perhaps pUS28 may contribute to tumour formation in these individuals.

The *UL33 *gene is conserved in all β herpesviruses, including HCMV (*UL33*), RCMV (*R33*), and MCMV (*M33*). While no ligands have been identified for either pUL33 or pR33, pM33 is activated by CCL5 [204]. All three viral proteins show constitutive activity, although they differentially activate specific signalling pathways [196,205-207]. While not essential for viral replication *in vitro*, both pR33 and pM33 are important for replication of CMV in the salivary gland [208-210]. In MCMV, this defect was linked to the constitutive activity of pM33 [211] and illustrates the importance of constitutive signalling *in vivo*. Like pUS28, pM33 and pR33 induce SMC migration [204,212]. Taken together, this suggests GPCRs of the *UL33 *family alter cellular trafficking during CMV infection, which may contribute to viral pathogenesis.

The *UL78 *gene family encoded proteins have only limited homology to known chemokine receptors [180]. Nevertheless, this gene is conserved in all β herpesviruses suggesting they have some role in the herpesviruses life cycle. The *UL78 *proteins of MCMV and RCMV, pM78 and pR78, respectively, are important for viral replication *in vitro *and *in vivo *[213-215]. The HCMV homolog, pUL78, is not required for replication of HCMV *in vitro *[216] thus MCMV and RCMV may be more dependent on its function for viral replication. Although, members of the *UL78 *family are clearly important for viral replication for some CMVs, data regarding their specific function as GPCRs is not currently available.

#### Viral Chemokine Binding Proteins

Chemokine binding proteins (CBPs) are virally encoded secreted proteins that competitively bind chemokines and interfere with their interactions with cellular receptors. Unlike viral chemokine and chemokine receptor homologues, which were likely acquired from the host, CBPs generally show no homology to known proteins and homology of these proteins across viruses is not conserved [217]. Until recently, no CMV species was known to encode a CBP. However, a HCMV encoded protein, p21.5, has been identified with chemokine binding properties. mRNA transcripts of the gene *UL21.5 *are packaged in the HCMV virion and thus may function soon after viral entry into the cell. Although many CBPs interact with multiple chemokines [218], p21.5 is unusual in that it selectively binds CCL5 (RANTES) *in vitro *blocking the interaction of the chemokine with its cellular receptors [219]. No data is available which evaluates the ability of p21.5 to interfere with CCL5 function *in vivo *and its effect on viral survival.

#### Apoptosis

Apoptosis is a mechanism for programmed cell death that plays an important role in the elimination of cells during development and virally infected cells as part of the host's innate immunity [220,221]. Many viruses including CMVs have strategies to prevent apoptosis [222,223]. HCMV encodes two proteins in particular that directly interfere with the apoptosis pathway. The HCMV *UL36 *gene encodes a viral inhibitor of caspase-8 induced apoptosis (vICA) and exon 1 of *UL37 *encodes the viral mitochondrial inhibitor of apoptosis (vMIA) [224].

vMIA protects cells from intrinsic apoptosis induced following damage to the mitochondrial membrane [225-227]. vMIA only blocks apoptosis mediated through the death receptor, Fas, in cells in which apoptosis proceeds through a mitochondrial-dependent step [228]. vMIA has not been identified in virion particles and inhibits apoptosis at later times compared to vICA [225,229]. vMIA localizes to the mitochondria [230-232] and this localization is needed for vMIA to induce structural changes and inhibit mitochondrial release of cytochrome c, blocking downstream events in the apoptosis pathway [227,233]. vMIA also induces the release of calcium from the ER which may play a role in the inhibition of apoptosis [234].

vMIA was initially shown to interact with adenine nucleotide translocator (ANT), a regulator of mitochondrial membrane permeability [227,235]. However, this interaction does not correlate with vMIA function and is likely non-specific [235]. vMIA also binds the proapoptotic protein, Bax, inducing aggregation of Bax molecules at the mitochondrial membrane preventing membrane permeabilization [236,237]. The screening of proteins that interact with the antiapoptotic domain of vMIA identified the protein, Growth Arrest and DNA Damage 45 (GADD45α). GADD45α enhances vMIA-induced apoptosis possibly protecting it from proteasomal degradation [238]. Interestingly, vMIA was also recently shown to interfere with caspase-independent cell death by blocking the activity of the serine protease, HtrA2/Omi [239]. Sequence homologues of vMIA have only been found in primate CMVs [240]. However, a recently identified positional homologue of vMIA in MCMV, m38.5, was shown to interact with Bax and inhibit apoptosis [226,241,242]. Therefore, vMIA functional homologues may exist in other cytomegaloviruses.

In contrast to vMIA, vICA protects cells *in vitro *from extrinsic apoptosis mediated through ligation of the death receptors, Fas and TNFR-1 [228]. vICA is a virion constituent [243] that inhibits caspase-8 activation [228]. Procaspase-8 is prevented from interacting with the adapter protein, Fas-associated death domain (FADD) and inhibits the processing of procaspase-8 to its active form [244]. Therefore, vICA is functionally similar, although mechanistically distinct from, cellular and viral FLICE inhibitory proteins (FLIPS), which also interfere with caspase-8 activation [245].

vICA is dispensable for replication and some HCMV lab strains have inactivating mutations in the *UL36 *gene [228,243,246]. Nevertheless, vICA homologues are conserved in the genomes of all sequenced β-herpesviruses except for guinea pig CMV (GPCMV) where no positional homologues has been identified [247]. The vICA homologues of RhCMV and MCMV, Rh36 and M36, respectively, also have antiapoptotic activities when transiently expressed *in vitro *[240,248]. Recently, it was shown that a M36 deletion mutant was attenuated in the lungs and salivary glands of mice but could be rescued by expression of a dominant-negative variant of FADD [249]. This provides important evidence for the functional significance of this protein *in vivo*.

The MCMV *M45 *encoded protein has sequence, although not functional, homology to a cellular ribonucleotide reductase [250,251]. Nevertheless, a M45 mutant virus is attenuated in SCID mice [251]. Recently M45 was shown to suppress the cell death pathway in a manner unique to viral proteins [252]. M45 interacts with the receptor-interacting protein kinase I (RIPI) via a RIP homotypic interaction motif (RHIM). By binding to RIPI, M45 protects certain cell types from caspase-independent cell death following death receptor signalling [253,254]. This "alternative apoptosis" would likely be activated in HCMV infected cells in which vICA inhibits caspase-8 activity [255].

### Adaptive Immune Response

#### T Cell Mediated Immunity

T cell mediated immune responses, particularly CD8^+ ^T cell dependent responses, are integral to the clearance of cytomegalovirus infections [54]. Cytomegaloviruses, as do many herpesviruses, impair T cell activation by interfering with both MHC class I and II antigen processing and presentation [256]. Although this has been shown *in vitro*, Th or CTL responses are still generated during CMV infection *in vivo *[257-260].

The U_S _region of the HCMV genome encodes a number of endoplasmic reticulum (ER) resident glycoproteins that alter MHC class I expression [261]. The first of these proteins to be expressed following HCMV infection is the *US3 *encoded immediate early protein, gpUS3, which likely interferes with T cell recognition early during viral replication [262-264]. gpUS3 binds MHC class I molecules and retains them in the ER, inhibiting antigen presentation to CD8^+ ^T cells [265-269]. The transmembrane domains of gpUS3 are responsible for binding MHC class I [270,271]. However, luminal regions of gpUS3 are needed for retention of MHC class I in the ER [270-273]. Interestingly, gpUS3 also binds tapasin and this interaction prevents tapasin-mediated protein loading of MHC class I molecules. Therefore, gpUS3 only retains MHC class I alleles that are tapasin dependent [274]. An alternatively spiced form of gpUS3 competitively binds tapasin and may represent a regulatory mechanism for gpUS3 activity [275].

In contrast to gpUS3, the binding of MHC class I molecules to the *US2 *and *US11 *encoded HCMV proteins, gpUS2 and gpUS11, leads to the rapid degradation of MHC class I molecules [276-279]. Both proteins bind MHC class I molecules resulting in their removal from the ER to the cytosol where they undergo proteasome-dependent degradation [266,276,277]. This process requires a functional ubiquitination system although only gpUS2 specifically depends on MHC class I ubiquitination for protein degradation [280-283]. In addition to the ubiquitin system, gpUS2 and gpUS11 associate with other cellular proteins that facilitate recognition and removal of proteins via the endoplasmic reticulum associated protein degradation (ERAD) pathway [284-287]. gpUS2 forms a complex with the chaperones, calnexin, BiP, and calreticulin to mediate MHC class I degradation [288]. In contrast, gpUS11-dependent protein degradation requires Derlin-1, a protein that plays a role in the removal of misfolded proteins [289].

Why HCMV encodes two different proteins that target MHC class I for degradation is unclear. gpUS2 and gpUS11 differ in their specificities for MHC class I alleles [290,291]. While they may overlap in function, they likely mediate distinct effects on MHC class I expression [292,293]. This difference in specificity is likely the result of different binding requirements for gpUS2 and gpUS11. The luminal portion of gpUS2 interacts with residues in the α_2_/α_3 _region of the luminal domain of MHC class I molecules [294-296]. Specifically, gpUS2-mediated degradation requires the presence of an arginine at residue 181 of MHC class I molecules. However, this residue is not sufficient for degradation of some MHC class I alleles and other residues must be important for these molecules [297]. The luminal region of gpUS11 is also important for MHC class I binding, however, gpUS11 interacts with residues in the α_1_/α_2 _domain of MHC class I molecules [298,299].

The HCMV *US6 *gene, encodes another glycoprotein, gpUS6, that downregulates MHC class I expression [121,300,301]. Like the other U_S _proteins that interfere with MHC class I expression, gpUS6 mediates retention of MHC class I via its luminal domain [302]. However, the gpUS6 mechanism is unique in that it binds to TAP-1 and TAP-2 heterodimers [303] in the transporter associated with antigen processing (TAP) complex [121,300,304,305]. Through its association with TAP, gpUS6 prevents the binding of ATP to TAP [306] and subsequent TAP peptide translocation [300,301]. gpUS6 expression is solely responsible for the inhibition of TAP in HCMV infected cells [307] and downregulates all MHC class I alleles tested [308]. In addition to impairing CD8^+ ^T cell responses, this decrease in MHC class I expression also makes infected cells susceptible to NK cell cytotoxicity [308], which may explain why CMVs have evolved mechanisms for preventing NK lysis (NK cell section).

Two additional HCMV encoded proteins, gpUS8 and gpUS10, bind MHC class I molecules. However, neither protein downregulates cell surface MHC class I expression, although gpUS10 slows MHC class I maturation and egress from the ER. The function of these proteins in HCMV infection is currently unknown [309,310].

Other cytomegaloviruses have homologues of some of the HCMV U_S _family members. CCMV encodes a gpUS6 homologue that binds TAP. However, this interaction does not downregulate MHC class I expression in chimpanzee cells *in vitro*, making the function of this homologue unclear [305]. In contrast, RhCMV encodes homologues of gpUS2, gpUS3, gpUS6, and gpUS11 [311], each of which functions similarly to its HCMV counterpart [312]. A newly identified RhCMV protein, viral inhibitor of heavy chain expression (VIHCE), is encoded by the *rh178 *gene, a gene unrelated to the US6 gene family. VIHCE inhibits signal-peptide dependent MHC class I heavy chain translation, a mechanism distinct from other modulators of MHC class I expression [313]. Therefore, RhCMV may be a valuable model system for analyzing the function of these proteins during CMV infection *in vivo*.

MCMV encodes three proteins unrelated to those of HCMV that interfere with MHC class I expression in infected cells. *m6*, *m152*, and *m4*, encode gp48, gp40, and gp34 respectively. Both gp40 and gp48 inhibit antigen presentation to CD8^+ ^T cells *in vitro *[314,315]. gp40 and gp48 bind MHC class I molecules and retain them intracellularly [314-316], although each protein does so via different mechanisms. gp40 retains MHC class I molecules in the ER-Golgi intermediate compartment (ERGIC)-cis golgi network [314] whereas gp48 binds to MHC class I molecules and targets them to lysosomes for degradation [315]. Deletion of *m152 *restores MHC class I expression in MCMV infected cells suggesting gp40 is the main regulator of MHC class I downregulation [317,318]. However, gp48 cooperates with gp40 to enhance MHC class I retention *in vitro *[319]. gp48 and gp40 also show different specificities for MHC class I alleles and thus are both required for efficient MHC class I downregulation in certain cell types [320,321]. Surprisingly, the presence of *m152 *does not impact the CTL response in mice based on studies using recombinant MCMVs. Therefore, the *in vivo *role of *m152 *as well as *m4 *and *m6 *is not clear [322-324]. One possible explanation for this lack of CTL alteration *in vivo *is uninfected antigen presenting cells (APCs) cross-priming CD8^+ ^T cells, which could circumvent function of gp40 in mice [324]. If these proteins cannot prevent priming of CD8^+ ^T cells, what is their function? There are a couple of possibilities. One is that the delay in CD8^+ ^T cell recognition is enough to allow the virus to establish a foothold within the host and eventually establish latency. Also without this initial CD8^+ ^T cell recognition, immunopathology could be diminished allowing the host to survive and viral spread. Recently the Reddehase group has shown that the presence of these immune modulating proteins (gp40, and gp48) actually enhance priming of CD8+ T cells [325]. How this benefits CMV survival *in vivo *is hard to reconcile, but may represent the evolution of mouse immune responses to counter these "immunevasions." The Reddehase and Hill labs are both actively persuing answers to these questions and why CMVs would have evolved mechanisms to enhance CTL priming.

The MCMV protein gp34 also associates with MHC class I molecules [326,327]. In contrast to gp40 and gp48, gp34 decreases the intracellular retention of MHC class I molecules [319,326]. However, this phenomenon is seen only in the absence of m6 (gp48) suggesting gp48 expression may antagonize the function of gp34 and provide a mechanism to regulate the extent of MHC class I expression in infected cells [317,319].

HCMV can also interfere with the presentation of MHC class II on antigen presenting cells such as macrophages [328]. gpUS3, gpUS2, and pp65 mediate this effect. gpUS3 binds and downregulates MHC class II, which subsequently decreases antigen presentation to CD4^+ ^T cells. gpUS3 interferes with the sorting of MHC class II to lysosomes inhibiting peptide loading in these compartments and subsequent MHC class II expression at the cell surface [269]. gpUS2 also interferes with CD4^+ ^T cell recognition by downregulating MHC class II molecules. Similar to its effect on MHC class I, gpUS2 binds the α chain of MHC class II resulting in the proteasome-dependent degradation of MHC class II molecules in a number of different cell types *in vitro *[269,329-331]. Lastly, the tegument protein, pp65, also downregulates MHC class II expression. Yet another mechanism for MHC class II down regulation, pp65 mediates MHC class II trafficking to lysosomes causing their destruction [332]. MCMV infection also downregulates MHC class II expression although the viral proteins responsible for this are not currently identified [141,333]. As observed with MHC class I downregulation, CMVs have evolved multiple modes for achieving manipulation of MHC class II host responses in order to achieve evolutionary success.

## Conclusion

Whether it is the destruction of important immune molecules, a redirection of these proteins intracellularly, transcriptional or translational control, alteration of signal transduction cascades, or the production of novel interfering proteins, CMVs "push" and "pull" at the immune response in such a way as to ensure their evolutionary success. Based on the numerous viral proteins that respond to the host's defence mechanisms, the relationship of CMVs with their hosts is complex. In spite of the manipulation of both the magnitude and quality of the innate and adaptive immune responses, the host still launches a robust anti-CMV immune response but not before the virus establishes latency within the host. Therefore CMV infection does not eliminate host immunity but modulates it to allow survival of both the virus and the host, as both are important to the life cycle of the virus. With its millions of years of co evolution within us, we can use CMVs acquired "knowledge" of the immune system to uncover novel immune pathways. We can potentially exploit CMVs potential vulnerabilities for developing CMV vaccines (i.e. ones that can not establish latency) or novel therapeutics that could minimize CMV-induced damage in immune compromised hosts.

## Competing interests

The authors declare that they have no competing interests.

## Authors' contributions

MMK carried out the literature review and wrote this manuscript. TES conceived of the review and edited the manuscript. All authors read and approved the final manuscript.

## Authors' Information

MMK recently received her PhD with a dissertation exploring CMV viral chemokines from the University of Tennessee. TES is an assistant professor at the University of Tennessee whose lab focuses on CMV immune modulation.

## Supplementary Material

Additional file 1 **Cytomegalovirus modulation of the innate immune response.** A listing of the cytomegalovirus encoded proteins that interfere with the innate immune response. Click here for file

Additional file 2 **CMV Modulation of the adaptive immune response**. A listing of the cytomegalovirus encoded proteins that interfere with the adaptive immune response. Click here for file
