# Supplementary material for: Feeling manipulated: cytomegalovirus immune manipulation
Source: Virol J. 2009 Jan 9;6:4. doi: 10.1186/1743-422X-6-4 (PMC2636769; doi:10.1186/1743-422X-6-4)
Supplement: Additional file 1 — Cytomegalovirus modulation of the innate immune response. A listing of the cytomegalovirus encoded proteins that interfere with the innate immune response. [file 1743-422X-6-4-S1.doc]

| **Table 1. CMV modulation of the innate immune response.** | | | |
| --- | --- | --- | --- |
| **Protein** | **Gene** | **Virus** | **Function** |
| **Complement Cascade** | | | |
| Unknown | Unknown | HCMV  MCMV | Complement resistance |
| **F_C_ Receptor Homolog** | | | |
| gp34 | TRL11/IRL11 | HCMV | Function unknown |
| gp68 | UL119-UL118 | HCMV | Function unknown |
| fcr-1 | m138 | MCMV | ↓ NK ligands H60 and MULT-1 |
| **Interferon-Mediated Immunity** | | | |
| IE1-p72 | IE1 | HCMV | Inhibits ISGF3 |
| IE86 | IE2 | HCMV | ↓ IFNβ expression |
| pp65 | UL83 | HCMV | ↓ ISGs |
| M27 | M27 | MCMV | ↓ STAT-2 |
| m142/m143 | m142/m143 | MCMV | ↓ PKR activity |
| TRS1/IRS1 | TRS1/IRS1 | HCMV | ↓ PKR activity |
| **Natural Killer Cells** | | | |
| gpUL18 | UL18 | HCMV | MHC I homolog; alters NK cell function |
| gpUL142 | UL142 | HCMV | MHC I homolog; ↓ NK cell function |
| pp65 | UL83 | HCMV | ↓ NK cell function |
| gpUL141 | UL141 | HCMV | ↓ CD155 expression |
| gpUL16 | UL16 | HCMV | ↓ NK cell function |
| miR-UL112 | UL112 | HCMV | ↓ NK ligand MICB |
| UL40 peptide | UL40 | HCMV | ↓ NK cell function |
| m157 | m157 | MCMV | MHC I homolog; ↑ NK cell function |
| m144 | m144 | MCMV | MHC I homolog; ↓ NK cell function |
| gp40 | m152 | MCMV | ↓ NK ligands of the RAE-1 family |
| m145 | m145 | MCMV | ↓ NK ligand MULT-1 |
| m155 | m155 | MCMV | ↓ NK ligand H60 |
| **Cytokine Homologs** | | | |
| cmvIL-10 | UL111 | HCMV | Induces anti-inflammatory response |
| RhcmvIL-10 |  | RhCMV | Induces anti-inflammatory response |
| **Cytokine Receptor Homologs** | | | |
| UL144 | UL144 | MCMV | Blocks T cell proliferation |
| **Viral Chemokine Homologs** | | | |
| vCXCL-1 | UL146 | HCMV | Activates neutrophils |
| vCXCL-1 | UL146 | CCMV | Activates neutrophils |
| pUL147 | UL147 | HCMV | Function unknown |
| pUL128-131 | UL128-131 | HCMV | Mediate viral entry/cell tropism |
| MCK2 | m129/131 | MCMV | ↑ salivary gland dissemination |
| pr131 | r131 | RCMV | ↑ salivary gland dissemination |
| GPCMV-MIP | Unknown | GpCMV | Activates HCCR-1 |
| **Chemokine Receptor Homologs** | | | |
| pUS28 | US28 | HCMV | Constitutive signaling; binds chemokine |
| pUS27 | US27 | HCMV | Function unknown |
| pUL33 | UL33 | HCMV | Constitutive signaling |
| pR33 | R33 | RCMV | Needed for salivary gland replication |
| pM33 | M33 | MCMV | Needed for salivary gland replication |
| pUL78 | UL78 | HCMV | Function unknown |
| pR78 | R78 | RCMV | Function unknown |
| pM78 | M78 | MCMV | Function unknown |
| **Chemokine Binding Proteins** | | | |
| pUL21.5 | UL21.5 | HCMV | Binds CCL5 |
| **Apoptosis** | | | |
| vICA | UL36 | HCMV | Inhibits extrinsic apoptosis |
| vMIA | UL37x1 | HCMV | Inhibits intrinsic apoptosis |
| m38.5 | m38.5 | MCMV | Inhibits apoptosis |
| M45 | M45 | MCMV | Inhibits death receptor signaling |
