# Supplementary material for: Feeling manipulated: cytomegalovirus immune manipulation
Source: Virol J. 2009 Jan 9;6:4. doi: 10.1186/1743-422X-6-4 (PMC2636769; doi:10.1186/1743-422X-6-4)
Supplement: Supplementary file 2 [file 1743-422X-6-4-S2.doc]

Table 2. CMV modulation of the adaptive immune response.

Protein	Gene	Virus	Function
**T Cell Mediated Immunity**			
gpUS3	US3	HCMV	Inhibits MHC I and II expression
gpUS2	US2	HCMV	MHC I and II degradation
gpUS11	US11	HCMV	MHC I degradation
gpUS6	US6	HCMV	Inhibits TAP peptide transport
gpUS8	US8	HCMV	Binds MHC I molecules
gpUS10	US10	HCMV	Binds MHC I molecules
pp65	UL83	HCMV	Inhibits MHC II expression
gp48	m6	MCMV	Targets MHC I to lysosomes
gp40	m152	MCMV	MHC I retention in the ERGIC
gp34	m4	MCMV	↓ MHX I ιντραχελλυλαρ ρετεντιον
